# Supplementary material for: Green Synthesis of Laser-Induced Graphene with Copper Oxide Nanoparticles for Deicing Based on Photo-Electrothermal Effect
Source: Nanomaterials (Basel). 2022 Mar 14;12(6):960. doi: 10.3390/nano12060960 (PMC8951176; doi:10.3390/nano12060960)
Supplement: Supplementary file 1 [file nanomaterials-12-00960-s001.zip › nanomaterials-1563347-supplementary.pdf]

nanomaterials

MDPI

Supplementary Material

Green Synthesis of Laser-Induced Graphene with Cu Oxide Nanoparticles for Deicing Based on Photo-Electrothermal Effect

Jun-Uk Lee [1,†], Jeong-hoon Lee [1,†], Chan-Woo Lee [1], Su-Chan Cho [1], Sung-Moo Hong [2], Yong-won Ma [2], Sung-Yeob Jeong [3] and Bo-Sung Shin [4,*]

1 Department of Cogno-Mechatronics Engineering, Pusan National University, Pusan 46241, Korea; lju3534@naver.com (J.-U.L.); kgslsk6219@naver.com (J.-h.L.); cwleeho2@naver.com (C.-W.L.); cho_brian@naver.com (S.-C.C.)
2 Interdisciplinary Department for Advanced Innovative Manufacturing Engineering, Pusan National University, Pusan 46241, Korea; hsm14789@naver.com (S.-M.H.); decentsoul@naver.com (Y.-w.M.)
3 Department of Mechanical Engineering, The University of Tokyo, Tokyo 113-8656, Japan; ysjsykj8025@naver.com
4 Department of Optics and Mechatronics Engineering, Pusan National University, Pusan 46241, Korea
* Correspondence: bosung@pusan.ac.kr; Tel.: +82-51-510-2787
† These authors contributed equally to this work.

Table S1 shows our specifications of the 355 nm UV pulsed laser.

Table S1. Specifications of the 355 nm UV pulsed laser.

Parameter	Unit	Value
Wavelength	nm	355
Average power	Watt	~1.5
Pulse duration	ns	25
Repetition rate	kHz	30
Mode	-	TEM_{00}
Beam diameter	mm	1.5
Beam divergence	mard	<0.5
Peak power	W	1466.67
Peak power density	W/cm^2	165992.72

Table S2 summarizes our laser beam condition (laser speed, spot size, dynamic fluence, overlapping factor).

Table S2. Laser beam condition.

Laser Speed (mm s^{-1})	Spot Size (μm)	Dynamic Fluence (J/cm^2)	Overlap Factor (O_f)
60	80	22.91	99.997
40	95	28.94	99.998

For a given laser power, P (W), and scanning speed, U (mm/s), a dynamic fluence, F (J/mm^2), can be defined as [54]:

$$F = \frac{P}{DU} \quad (1)$$

where D [mm] is the laser beam spot diameter. The product DU in the equation corresponds to the area exposed by the effective focal spot as the laser head moves along a predefined path.

Overlapping factor can be calculated as [55]:

$$O_f = \left(1 - \frac{v/f}{D + vt}\right) \times 100 \quad (2)$$

where O_f is the overlapping factor, D is the laser beam diameter on the workpiece, t is the pulse duration (ms), f is the pulse frequency (Hz), and v refers to the laser speed (mm/s).

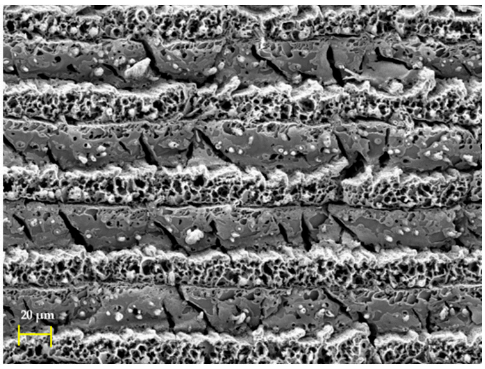

Figure S1. FE-SEM image of LIG fabricated with 0° hatching process.

It can be seen that only the laser-irradiated portion is formed with a porous LIG. Line spacing is 50 µm.

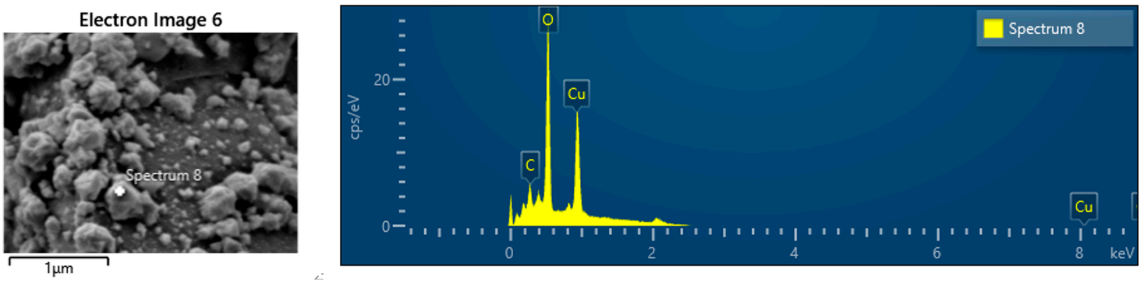

Figure S2. EDS spectra of Cu/LIG.

For further analysis, we conducted extreme EDS analysis to check if dehydrated copper chloride is present. Our eds spectra show Cl peak does not exist on the Cu oxide nanoparticle's surface.

Table S3. EDS spectra of Cu/LIG.

Element	Line type	Apparent concentration	Wt%	Wt% sigma	Atomic %	Standard Label
C	K series	0.13	7.51	0.39	17.02	Pure element
O	K series	0.98	34.04	0.41	57.93	SiO2
Cu	L series	1.38	58.46	0.48	25.05	Pure element
Total:			100.0		100.0	

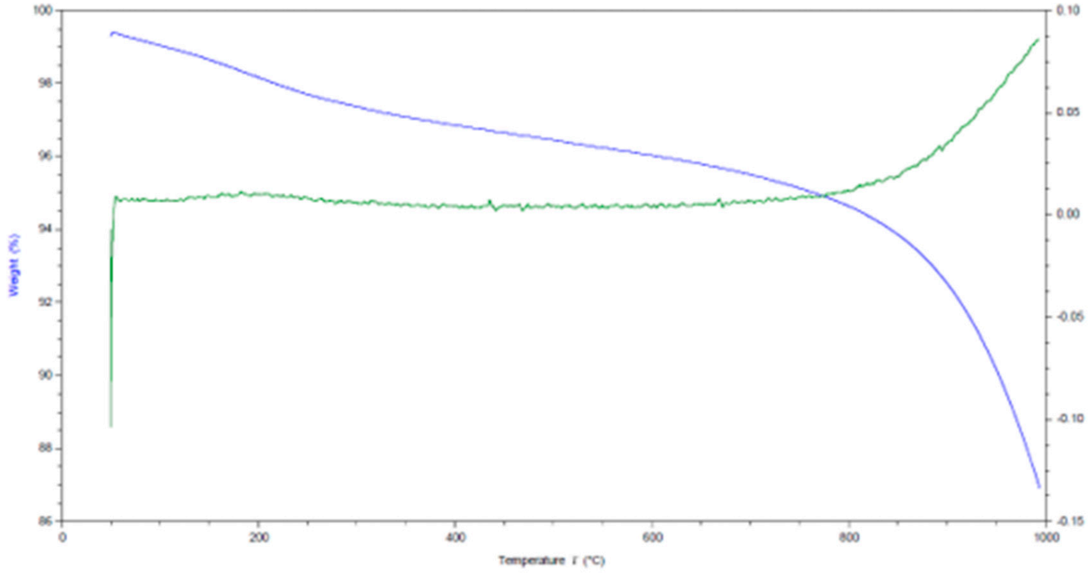

Figure S3. TGA curves of Cu/LIG.

We confirm that copper oxide nanoparticles can enhance thermal stability through TGA analysis. Initial weight loss until 200˚C caused by moisture vaporization.

In the previous research, bare LIG shows a rapid weight loss because of relatively weak functional groups [49,56]. Functional groups are dissipated around 550 ˚C. The LIG remained 60 w% at 800˚C. Copper oxide nanoparticle-enhanced LIG still remains 97 w% until 550˚C. Carboxy or hydroxy functional groups with weak binding force are gradually dissipated. Finally, it can be seen that 87 w% is maintained at 1000 ˚C.

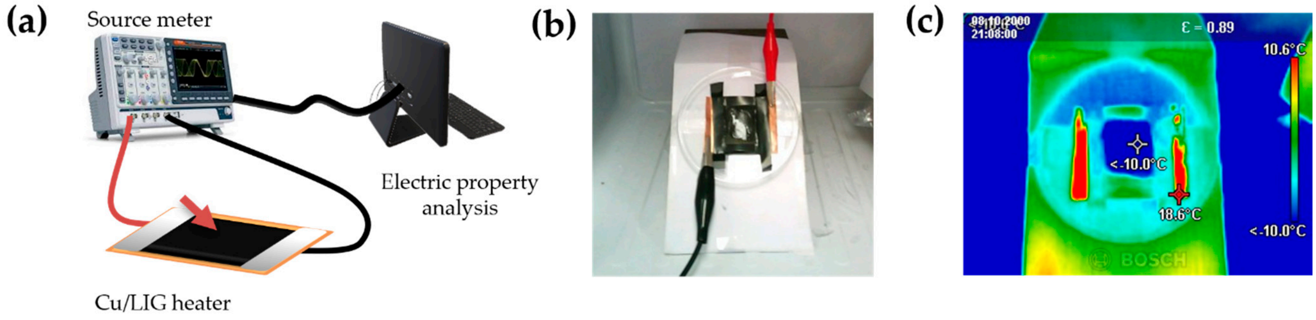

Figure S4. (**a**) Illustration of electrothermal performance test for deicing, (**b**) actual photograph of Cu/LIG with ice accumulation of the surface, (**c**) thermal images of Cu/LIG patterns with ice accumulation.

Figure S4 shows a schematic illustration of electro heating performance for deicing. We conducted an experiment under 10˚C for practical application. The actual experimental setup is shown in Figure S5 (b). We applied a voltage of 10 V on Cu/LIG with 3 X 3 cm^2. Figure S5 (c) shows the thermal image; the power density was measured to be about 1.06 W/cm^2.
